# Representation, attribution, and purification: a study on the dissemination of competitive sports public opinion in the digital media environment

**DOI:** 10.3389/fsoc.2025.1591882

**Published:** 2025-08-29

**Authors:** Yan Yang, Yueting Zhang, Bing Shi

**Affiliations:** ^1^Physical Education and Research Department, Shangluo University, Shangluo, Shaanxi, China; ^2^Physical Education School, Shaanxi Normal University, Xi’an, Shaanxi, China

**Keywords:** competitive sports, digital media, network public opinion dissemination, representation, attribution, purification

## Abstract

Taking the dissemination of competitive sports public opinion in the digital media environment as the research object, this study adopts case analysis, phenomenology, narrative evaluation, and logical induction to examine the phenomenon from the perspective of communication characteristics. It rationally analyzes the manifestations and causes of communication misconduct and proposes purification strategies to promote the high-quality dissemination of competitive sports public opinion culture. The study finds that the phenomenological essence of competitive sports public opinion dissemination under digital media is characterized by an “information dividend” resulting from explosive growth, which accelerates the transformation of athletes into internet celebrities. Symbolic communication enhances the visibility of competitive sports-related public opinion while simultaneously encouraging the proliferation of peripheral behavioral subcultures. Interactive marketing-driven dissemination contributes to the precise identification and recommendation of market needs. However, this also gives rise to issues such as online public safety risks, forced negative intervention in athletes’ daily life, training, and competitions, as well as the chaotic and uncontrollable spread of public opinion. Drawing on Actor-Network Theory (ANT), this study constructs a tripartite attribution framework—actor, translation, and network—to explain the causes of misconduct. These include athletes’ personal expression, role-playing needs, and self-presentation desires; public idol worship; the rise of digital consumer culture; and increasing societal demand for sports public figures. To purify the public opinion environment, the study proposes a comprehensive approach based on moral constitutional governance, emotional guidance, and collaborative governance. This involves strengthening moral and legal systems governing online discourse, enhancing the guiding and regulatory role of mainstream media, and constructing a collaborative governance mechanism involving multiple communication actors.

## 1 Introduction

With the advent of the information technology revolution represented by the Internet, people’s work, life, behavior, and modes of information acquisition have undergone fundamental transformations (Lee et al., 2018). Traditional media, such as newspapers, radio, and television, are facing significant challenges in the digital media era (Salman et al., 2011; Hassan et al., 2021). Leveraging digital technologies and network-based platforms—including computers and mobile devices—digital media has constructed a distinctive cultural ecosystem for information dissemination. Platforms such as self-media, social media, and short video services have emerged as new modes of communication, attracting broad public engagement (Kuang and Liu, 2023). Since 2020 in particular, the emergence of the Web 3.0 era—characterized by decentralization and intelligence—has ushered in a new phase in the evolution of digital media. Within this context, the dissemination of public opinion related to competitive sports has primarily centered on controversies surrounding sporting events or highly attention-grabbing individuals and socially relevant incidents. These discussions are amplified by a vast networked social circle composed of self-media influencers, internet celebrities, and fan-based online communities (Salman et al., 2011; Wu et al., 2023). Within this virtual social circle, all athletes are endowed with the role of subjects and carriers of competitive sports network public opinion dissemination, thereby breaking the traditional paradigm of sports star–centered dissemination. The idolization of ordinary “civilian” athletes on the internet has become a prevailing phenomenon. As idols are demystified, athletes are increasingly perceived as symbolic representatives of sports culture, with individual labels replacing collective identities. Consequently, the athlete-driven portrayal of sports images exerts a significant influence on public perceptions and behavioral orientations toward sports. Particularly under the discourse model dominated by a relatively liberal online public opinion environment, athletes are increasingly exposed and hyped as symbolic vessels of commercial capital. This trend metaphorically shifts the public’s focus toward events peripheral to athletic competition while neglecting the intrinsic value and purpose of competitive sports itself (Xu and Wang, 2022). In the current context of China’s competitive sports public opinion dissemination, the excessive “internet celebrity-ization” of athlete stars reflects an overwhelming pursuit of entertainment effects. In online spaces, the rampant infringement of athletes’ personal privacy has become normalized. Driven by self-interest, fan-based behaviors such as support rankings, manipulated traffic metrics, targeted searches, cyberbullying, and antagonistic fan group conflicts have emerged as common cultural phenomena in the dissemination of sports-related public opinion. This “fandom-ization” of the digital public sphere poses serious risks to the personal development of athletes and is profoundly detrimental to the overall advancement of competitive sports (Xiaowei, 2025; Yu et al., 2019).

In response to the phenomenon of alienation in the dissemination of competitive sports public opinion within the digital media environment, scholars both domestically and internationally have made significant breakthroughs. Representative research findings in this field can be broadly categorized into the following areas: (1) The influence of online public opinion dissemination on competitive sports in the digital media environment: Yu et al. analyzed the disruptive impact of mobile-based sports media dissemination on sports-related public opinion. Their study explored the structural composition and polarization effects of online sports public opinion and proposed corresponding strategies and pathways for guiding public discourse in the digital age (Yu et al., 2019). Johnson highlighted the ways in which racialized fan violence, when disseminated through media channels, affects Black athletes, revealing the critical role fan media plays in both shaping athletic images and reinforcing racial bias (Johnson, 2020). Gong examined the mechanisms underlying the formation of online public opinion and its implications for sports management, emphasizing the growing importance of digital platforms as spaces for ideological exchange in modern society (Gong, 2017); (2) Socio-phenomenological interpretations of public opinion dissemination in competitive sports: Forde and Wilson investigated the role of “sports media activism” in addressing social issues related to sports. They analyzed the participation strategies, motivations, and goals of sports media activists across various media contexts, offering insight into how activism is manifested in sports journalism (Forde and Wilson, 2018). Cao explored the complex interrelationship between sports and politics in the context of online public opinion. His research underscored the importance of the Olympic spirit in promoting human harmony and contributing to the construction of a peaceful society (Cao, 2022); (3) Research on the governance of online public opinion and dissemination strategies in competitive sports: Wan et al., through a case study of the Chinese women’s volleyball team’s victory in the 2019 World Cup, proposed a dual-perspective framework—based on network structure and emotional expression—to analyze the evolution of online public opinion surrounding sports events. They further introduced new conceptual approaches to public opinion governance based on this analytical lens (Wan and Chen, 2021). Xie et al. examined the dissemination mechanisms, media roles, and multidimensional environments of public opinion in the context of emergencies related to competitive sports. Based on their findings, they advocated for interdisciplinary strategies to enhance public opinion monitoring and response capacities (Xie et al., 2022). Alqmase et al., within a digital media framework, constructed sentiment classification models using emotion analysis techniques to automatically identify and categorize superstitious and anti-superstitious sentiments in Arabic social media, thereby promoting the healthy dissemination of competitive sports-related public opinion (Alqmase et al., 2021). Wang et al. analyzed the dual role of short-form videos in the dissemination of sports public opinion, exploring both their benefits and drawbacks. They proposed targeted improvement measures and monitoring systems to optimize information pathways in sports-related public opinion dissemination (Wang et al., 2021). Huang et al. employed Python and grounded theory methods to investigate the evolution mechanisms of online public opinion during emergency incidents in major sporting events. Their research revealed the phased characteristics and primary influencing factors of such public opinion, while offering corresponding governance and response strategies (Huang et al., 2024). Yang studied the evolution process and influencing variables of online public opinion during the 2022 Beijing Winter Olympics under the backdrop of big data, providing both theoretical insights and practical recommendations for the governance of sports event-related online public opinion (Yang, 2023). Du analyzed the public opinion crisis during the Tokyo Olympics, exploring the patterns of development and dissemination in such crises and proposing appropriate response strategies (Du, 2023). Li et al. examined the impact of online public opinion in the new media era on the brand image of sporting events, analyzing both its positive and negative effects, and recommending monitoring and countermeasure strategies tailored to the online media environment (Li et al., 2021). Wang investigated the dual influence of online public opinion on the communication of athletes’ images, analyzing the internal tensions between these dynamics and proposing strategies to preserve athletes’ reputations in the face of networked public discourse (Wang, 2014).

Despite the growing body of research on the dissemination of competitive sports public opinion in the digital media environment, existing literature remains largely focused on three primary dimensions: the impact of online public opinion on competitive sports, socio-phenomenological interpretations of public discourse, and governance or communication strategies related to online sports public opinion. However, several critical gaps persist. First, much of the current research adopts a single-dimensional analytical perspective, primarily emphasizing dissemination mechanisms and response strategies, while offering limited in-depth analysis of the representational content of public opinion. As a result, the symptomatic manifestations of problems in the dissemination process of competitive sports public opinion remain underexplored. Second, although some scholars have addressed the emergence of various sociocultural issues within the context of online sports discourse, there remains an overall lack of comprehensive attribution analysis within a broader social and cultural framework. Third, while existing studies often highlight the negative effects of public opinion dissemination, few provide concrete and actionable measures aimed at purifying the digital space and cultivating a more positive and healthy public opinion ecology. The dissemination of competitive sports public opinion is not merely a matter of content generation and diffusion—it also involves the systematic reduction of negative sentiment and the restoration of fairness and objectivity in public discourse. Therefore, under the current digital media landscape, this study not only focuses on the representational aspects of public opinion content and the sociocultural meanings it conveys but also explores the multidimensional mechanisms that drive public opinion formation. Moreover, it proposes strategies for purifying online discourse in order to construct a more rational and equitable ecosystem of sports public opinion. This research seeks to contribute to the creation of a cleaner and more constructive environment for the dissemination of competitive sports culture in the digital sphere. It aims to support the sound development of digital-era sports communication, regulate and guide the online behavior of professional athletes, and foster a positive and well-ordered online atmosphere. Ultimately, this study offers new theoretical support and practical guidance for the governance and optimization of digital sports public opinion, serving the broader goals of enhancing competitive sports and athlete development in China.

## 2 A phenomenological examination of competitive sports online public opinion dissemination in the digital media environment

Online public opinion in the realm of competitive sports refers to the aggregate of viewpoints, opinions, and emotions expressed, disseminated, and interacted with by the public regarding various competitions, figures, and events within the competitive sports domain through digital platforms such as news websites, online forums, and personal social media accounts. Under the digital media environment, the dissemination of public opinion related to competitive sports is characterized by a high degree of openness, freedom, virtuality, and interactivity. Individuals are able to access diverse streams of information, express their views, and engage in discussions through multiple channels. This participatory structure facilitates a shift in the role of public participants—from marginal observers to central actors—thereby accelerating the rapid spread and amplification of online public opinion. Phenomenology, a significant school of thought founded by German philosophers, is a research method that emphasizes the “direct understanding” of phenomena. It seeks to uncover the essence of a phenomenon through the lived experiences of participants, offering a powerful lens through which to interpret human consciousness and experience (Editorial Committee of Dictionary of Foreign Philosophy, 2008). Applying this framework, the dissemination of competitive sports public opinion in the digital media environment can be interpreted as a socially pervasive phenomenon. Through phenomenological inquiry, the study aims to reveal deeper insights into the essence of this phenomenon, going beyond surface-level descriptions to understand its underlying structural and experiential dimensions.

### 2.1 The explosive growth of online public opinion dissemination can generate information “dividends”

In traditional offline societies, the dissemination of competitive sports information primarily relied on word-of-mouth or written transmission. The carriers of dissemination, such as the human brain or paper materials, were relatively fixed, resulting in limited dissemination range, low efficiency, and slow speed (Peng, 2022). The intervention of digital media has transformed the traditional offline information dissemination ecology. The involvement of algorithms has qualitatively changed the storage methods, dissemination range, efficiency, and speed of athletes’ personal information. Information recipients can also use algorithms to process, analyze, and control athletes’ information, thereby generating various digital products. The fission effect generated by digital products is the root cause of the explosive growth of online public opinion information, which inadvertently enhances the economic value of information. Consequently, the dissemination of information through online public opinion has naturally become a tool for pursuing “dividends.” With the increasing influence of sports events, the rapid growth in attention to various online public opinion information about athletes has led to a surge in endorsements and advertisements. According to Forbes magazine’s list of the top ten endorsement incomes in global sports over the past year, published on May 11, 2022, Federer topped the list with $90 million, followed by LeBron ($80 million) and Messi ($55 million). Additionally, Gu Ailing, as a beneficiary of the “dividends” from the online public opinion dissemination of the Beijing Winter Olympics, signed contracts with 26 domestic and international brands in a short period, with endorsement value reaching $35 million. The aforementioned phenomenon of information “dividends” in competitive sports is an inevitable outcome of digital economic development. It has given rise to new models that support the high-quality development of competitive sports, marking the advent of a digital sports industry and an era of digitalized sports consumption. However, athletes’ excessive pursuit of such information-driven dividends, along with the over-commercialization of online public opinion surrounding star athletes by sponsors, has led to the growing trend of athlete “internet celebrity-ization.”

### 2.2 Leveraging symbolic communication to enhance online public opinion visibility

Symbolization refers to the process of transforming abstract ideas, emotions, experiences, or real-world phenomena into more comprehensible, communicable, and shareable forms through the use of symbols such as text, images, or sound (Li, 2024). In the context of digital media, the symbolic communication paradigm in the dissemination of competitive sports public opinion involves converting information related to competitions, athletes, and events into symbolic representations. These representations are then transmitted within the digital information space as cultural symbols that facilitate understanding, dissemination, and engagement, thereby enhancing the visibility and communicative efficacy of online public discourse. Athletes, as the external carriers of symbolic communication in competitive sports, become focal points in this process. By transforming athletes’ personal behaviors into distinctive cultural symbols and disseminating them in digital spaces—or by labeling them with attention-grabbing descriptors—media and users increase their appeal. This enables such athletes to rapidly become central figures and trending topics within the online public opinion culture of social communities (Zhang, 2017). For instance, during the 2023 Hangzhou Asian Games, numerous self—media accounts promoted Wu Yanni’s “signature moves,” and video accounts like Global Times and Dongqiudi reported under titles such as “Wu Yanni Responds” and “Performative Athlete Wu Yanni,” with each report exceeding 100,000 views. “Wu Yanni False Start (10.136 million)” and “Wu Yanni’s Result Canceled (6.502 million)” dominated the top two spots on the hot search list on the night of the finals, while “Lin Yuwei’s 100 m Hurdles Gold Medal (1.773 million)” only ranked seventh. The strong symbolic dissemination of athletes’ behaviors quickly made them sports internet celebrities, reflecting an underlying neglect of the essence of competitive sports culture. Disseminators attempt to provide more hot topics for online public opinion debates in the digital media space, aiming to radiate and derive more “other” behavioral cultures to increase public opinion heat.

### 2.3 Interactive dissemination of online public opinion facilitates the precise identification and targeted promotion of sports marketing audiences

Interactive dissemination of online public opinion refers to the collection of viewpoints, opinions, and emotions expressed, transmitted, and exchanged by the public via internet platforms such as social media, news websites, and online forums (Wang, 2025). This communication process emphasizes both interactivity and immediacy. During interactions, audiences simultaneously act as information producers and receivers, forming bidirectional or multidirectional communication patterns within the digital space. In the digital media environment, the interactive dissemination of public opinion in competitive sports has redefined the role of traditional media. For instance, Five Star Sports leveraged Taobao Live’s “Five Star Guest Room” to broadcast an intra-squad blue-white match of the Shanghai Shenhua football team using a split-screen format, simultaneously livestreaming the game and promoting products. Through fan-host interaction in the online public opinion space, the event achieved a dual win: increasing both sports event influence and product brand visibility. Notably, the second livestream on May 12, 2020, attracted over 240,000 viewers, boosted followers by 67%, and reached more than 14.6 million users, with Shenhua fans emerging as a dominant consumer group. Moreover, digital media can harness advanced digital technologies such as big data and cloud computing to conduct comprehensive tracking of public opinion content during sports events and livestream marketing sessions. By analyzing user preferences, product dwell times, and viewing timeframes, these data can be used to precisely identify and target marketing audiences. Such data-driven approaches significantly enhance the efficiency and accuracy of marketing strategies.

## 3 Representations of misconduct in competitive sports public opinion dissemination in the digital media environment

The concept of representation is widely employed in the field of communication studies. According to Glass, representation refers to “a mode of recording or expressing information,” that is, the reproduction of a particular object or concept through symbols, language, images, or other forms (Glass et al., 1986). In today’s digital media era, where online platforms are pervasive, people increasingly use mobile phones and computer applications to consume short videos and livestreams as a preferred form of leisure and time use (Ke et al., 2018). The rapid and high-quality development of the digital sports economy has become a key driving force in the transformation of the competitive sports industry. This phenomenon is reflected in the rise of livestream-based marketing and virtual gifting by internet influencers, which generate considerable economic returns. When such practices are extended into the competitive sports domain, some professional athletes or affiliated stakeholders exploit digital platforms—often employing vulgar or lowbrow entertainment tactics—to manufacture online public opinion. These marketing strategies, driven by the pursuit of online influence, visibility, and attention, contribute to the rise of the “internet celebrity economy” within competitive sports. While this may reflect a new norm in the development of competitive sports under digital conditions, the widespread nature of such behavior inevitably leads to both positive and negative consequences. How should this phenomenon be understood? It is necessary to approach the current enthusiasm surrounding the dissemination of online public opinion in competitive sports with critical reflection. In doing so, we can rationally assess the symptomatic representations of communication misconduct and provide informed, balanced responses to these emerging challenges.

### 3.1 The lack of moral and legal constraints in public opinion empathy expression induces network public safety crises

From the perspective of communication studies, public opinion is defined as “the sum of beliefs, attitudes, opinions, and emotional expressions of the public regarding various phenomena and issues in real society” (Wang, 2019). In the digital media environment, competitive sports news reporting has achieved a high degree of integration between online and offline life, as well as virtual and physical reality. The high—frequency, virtual “remote presence” has reached an interactive effect equivalent to “real presence.” As an important component of public opinion, the online dissemination of sports hotspot events helps people express their beliefs, attitudes, opinions, and emotions through digital media. Media often prioritize “empathic” expressions in news narratives to better create a public opinion atmosphere and bridge the emotional distance between the audience and the reported events. For example, in the news dissemination of Olympic champion Wang Liping, highly focused titles in online public opinion included “The Loneliest Olympic Champion Wang Liping,” “Deeply Moving Wang Liping,” “The Power of Never Giving Up,” and “Abandoned by the Coaching Team After Winning the Championship Without Draping the National Flag.” These reports, infused with emotional factors, control the discourse power in the public opinion field through the online popularity of sports stars, attracting widespread attention from social groups. Under the empathic dissemination mechanism, group entry behavior has achieved the rapid spread of sports culture, and online public opinion discourse has become a cloud—based emotional expression for the masses. However, the content of public opinion emotional expression mainly focuses on support, encouragement, moving, and sadness for the champion’s glory, as well as criticism of the coaching team’s negligence. This manifests as two tendencies: positive encouragement and online violent attacks after group entry. Particularly, the report “Abandoned by the Coaching Team After Winning the Championship Without Draping the National Flag” triggered online public opinion ranging from criticism of the coaching team’s incompetence, dismissal, accountability, and apology to lowbrow abusive language. The escalating violence in the public opinion field, driven by false exaggeration, incitement of opposition, distorted interpretation, and value distortion by disseminators, impacts the cultural order of online public opinion. The emotional “empathy” system is replaced by “ruthless” self—production. Once it dominates the public opinion field, it can easily induce a crisis in network public behavior safety.

### 3.2 Over-exploitation of commercial value disrupts athletes’ normal life, training, and competition

Driven by the interests of the digital influencer economy, online consumerism prevails. Online public opinion, as a “scarce” information resource, has advantages that traditional commodity economies cannot match, achieving maximum benefits through rapid dissemination in a short period (Dumlao, 2006). Transforming sports stars into online opinion leaders to enhance their commercial value has become a popular approach among stakeholders in competitive sports in the digital media era (Meng and Zou, 2023). Particularly leveraging the influence of major sports events, social commercial capital intervenes, packaging sports stars through online public opinion before, during, or after the events. This includes live streaming, endorsements, and participating in variety shows, excessively exploiting individual commercial value, which has become a common phenomenon in competitive sports.

Reviewing typical cases of the inception of the influencer economy in Chinese competitive sports, it can be said that since the 2016 World Table Tennis Championships in Kuala Lumpur, where both men’s and women’s teams won championships, efforts have been made to enhance athletes’ online influence and dissemination power. Athletes have been shaped into symbolic commercial images for media promotion, such as “The Fierce Tiger of the Empire” Zhang Jike, “The Dragon of Destruction” Ma Long, and “The Demon King” Zhang Yining. During the 2016 Rio Olympics, many sports stars “broke out” of their traditional roles. The star effect combined with the hot spots of major sports events allowed social entertainment capital to quickly turn them into subjects of online public opinion without much image packaging. Examples include “The Wild Girl” Fu Yuanhui, “The Asian Flying Man” Su Bingtian, and “The Big Sister” Yan Ni. Major media outlets, besides symbolizing athletes’ identities, deeply explore athletes’ growth experiences and daily lives. Using an “entertainment—first” extreme action logic to promote online identities, having athletes participate in various variety shows to increase exposure has become a norm. While athletes’ individual commercial value is enhanced, the lack of scientific management and normative guidance for athletes’ behavior leads to excessive entertainment consumption by the market, forming a strong counterproductive force against the development of competitive sports. This constitutes an internal driving force for the occurrence of deviant behavior. The paradigm of pan—entertainment thinking gives rise to a crisis of deviant behavior in online public opinion dissemination. Disseminators attempt to influence athletes’ normal life, training, and competition with a voyeuristic mindset, necessitating vigilance against the pressure and risks brought by the “over—consumption” of athletes’ physical and mental well—being by non-competitive factors.

### 3.3 Profit-driven utilitarian thinking causes chaos and uncontrollability in online public opinion dissemination

The advent of digital media has ushered in a prosperous era for the self-media industry. As the main body of cultural dissemination in the field of competitive sports, self-media can freely release and express individual or group sports cognition, attitudes, emotions, and behavioral intentions in cyberspace. Dissemination activities are placed in a relatively free, open, and tolerant virtual space, increasing the complexity of online public opinion management work. Various public opinion crises and risks are lurking. From the government’s perspective, there has always been an emphasis on media adhering to both economic and social benefits. However, with the deepening of the market economy, the profit-driven utilitarian thinking of self-media has apparently weakened its social effects. Disseminators, to cater to the audience and gain clicks and attention, publish some low-taste, gossip news reports. The image of competitive sports is hidden in the arbitrary comments and mutual attacks of fan groups. Under this dissemination thinking, the chaos and uncontrollability of online cultural dissemination are manifested in several ways: disseminators’ deviant speech infringing on others’ privacy, extreme personal expressions triggering online public opinion, false information confusing the truth of events, rumors being infinitely amplified, internet trolls disrupting online order for profit, and “three vulgarities” content inducing some irrational behaviors. For example, in May 2024, a self-media outlet published a news report titled “60 Days Countdown to the Paris Olympics, President Macron Wants to Strike.” At first glance, the title suggests that the president wants to refuse to host the Olympics, but the actual content disseminated includes: workers striking for bonuses, severe pollution of the Seine River affecting marathon swimming and triathlon events, security personnel shortages, and China’s “Olympic fever.” The report contains no information related to the host country’s president. Additionally, searching for the hot event “Asian Cup” on WeChat video accounts, online public opinion dissemination about the national football team is mostly focused on negative criticisms such as “disband,” “sure defeat,” “shame,” “incurable,” and “dismissal.” People have shifted from idol worship to collective online public opinion attacks. This irrational value orientation forming public opinion hegemony cannot fundamentally discover or solve the internal problems of the national football team. The fanatic guardianship of online public opinion dissemination and the chaotic order of irrational behavior are not conducive to the construction of the national sports image.

## 4 Attribution of misconduct in competitive sports public opinion dissemination in the digital media environment: a perspective based on actor-network theory (ANT)

**Figure 1:**
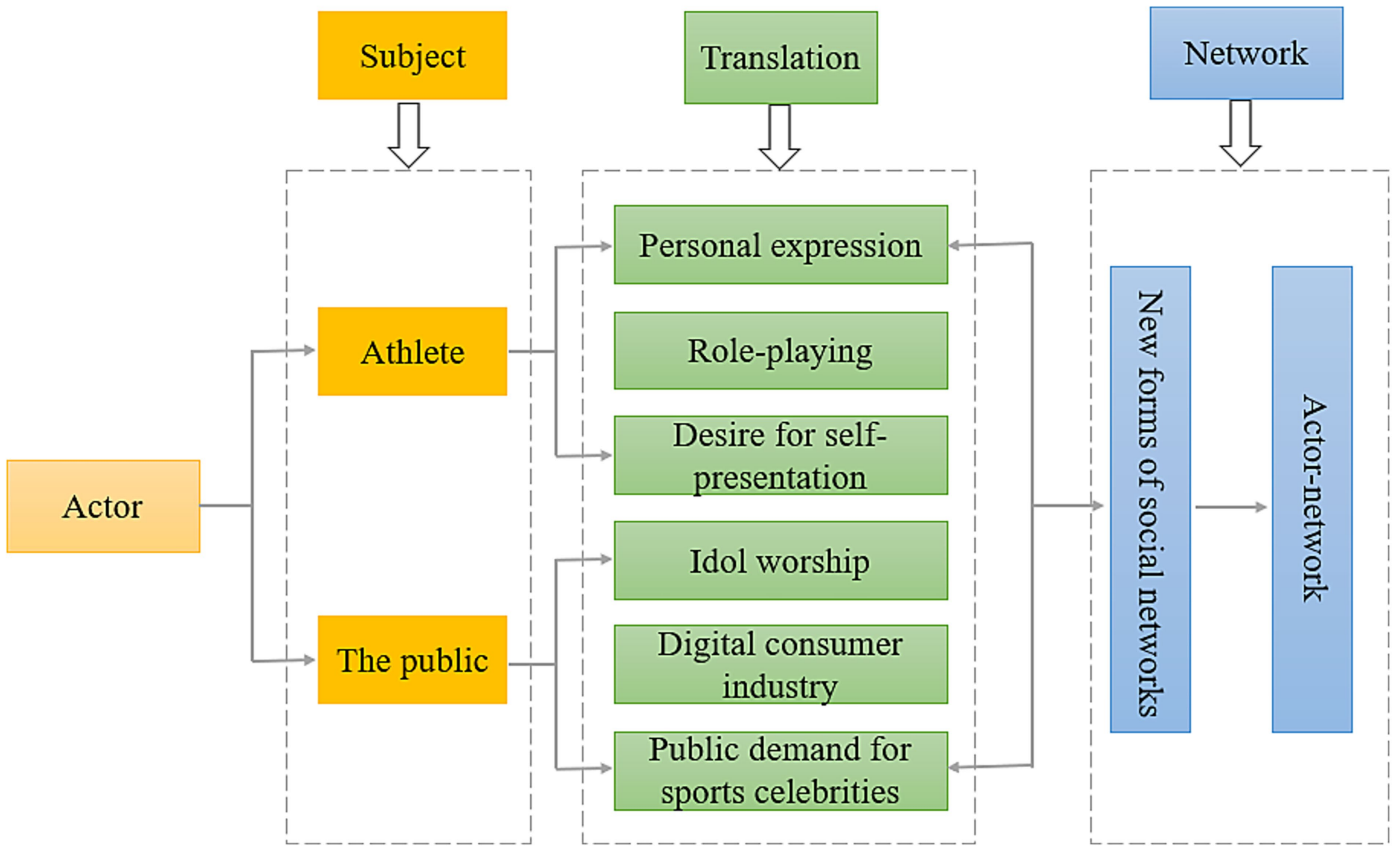
Analytical framework for the attribution of misconduct in competitive sports public opinion dissemination from the perspective of actor-network theory (ANT).

*Attribution* refers to the process by which individuals interpret and infer the causes of behaviors, events, or phenomena—either of themselves or others. This process helps individuals understand underlying motivations and better predict or regulate future behaviors (Zhang and Jin, 2008). In this study, the attribution of misconduct in the dissemination of competitive sports public opinion within the digital media environment is examined through the lens of Actor-Network Theory (ANT). Actor-Network Theory (ANT), developed in the mid-1980s by French sociologists Bruno Latour and Michel Callon, is a sociological framework that analyzes the formation of social phenomena through the interactions between human and non-human actors (Jiang and Zhang, 2025). To explain the widespread challenges in online sports public opinion—such as public safety risks, the psychological and physical overexploitation of athletes, and the chaotic and uncontrolled nature of online discourse—this study constructs a theoretical attribution framework based on ANT. The framework includes three interrelated components: actors, translation, and networks (see Figure 1). In the context of ANT, *actors* refer primarily to athletes and the general public, but also include coaches, event organizers, and service personnel. Each actor plays a distinct role in the online public opinion space. Athletes, through personal expression, role-playing, and the release of self-presentation desires, engage in the *translation* of individual information into online discourse. On the other hand, the public’s behaviors—driven by idol worship, the rise of the digital consumer culture industry, and the societal demand for sports celebrities—transform athletes’ information into widely disseminated public opinion. Together, athletes and the public serve as the primary agents of translation in the dissemination process, and their continuous interaction forms a new type of social structure: the *actor-network system*. The following section presents a detailed attribution analysis of the misconduct in competitive sports public opinion dissemination in the digital media environment, grounded in the core components of Actor-Network Theory.

### 4.1 The role-playing needs of athletes and the rise of the mass digital consumer culture industry

In the era of traditional media, athletes, as symbols of competitive sports culture creators, relied on newspapers, magazines, radio, and television to disseminate sports culture, the “positive hero image” of athletes became the core of promotional packaging (Mu, 2022). With the advent of the digital media era, especially the rise of self—media, the widespread dissemination of athletes’ public opinion driven by the digital economy has transformed the singular identity of competitive sports culture creators. Athletes now embody dual roles as both creators and disseminators of culture. This shift has enhanced the commercial value of athletes, particularly with the intervention of network capital, which has driven the transition of athletes’ role—playing needs from creators to disseminators. The intrinsic cultural essence of competitive sports carried by athletes has been weakened, while their social identity symbols have gained widespread recognition. Athletes have become prominent figures in various variety shows and social media platforms.

The dissemination of public opinion in the field of competitive sports attracts the joint attention of capital, media, and fans (Bernstein and Blain, 2002; Dart, 2014). The promotion of athletes’ identities has shifted from the pursuit of “positive hero images” to the exploration of off—field information. Driven by both exposure and economic benefits, this has led to the entertainmentization of competitive sports. Athletes gain network exposure and attention to enhance their roles as disseminators, measured by viewership, clicks, comments, and shares. This shift transforms media value into economic value, promoting the rise of a new type of mass digital consumer culture in the digital media era. This consumer culture industry follows the commercial logic and rules of the digital economy market, aiming to please the public’s sense of pleasure and participation. It creates a network public opinion cultural ecology that turns athletes into network cultural commodities, leveraging public opinion dissemination to meet the public’s network consumption needs for competitive sports. Once athletes, as dual—role creators and disseminators of sports culture, enter the commercial realm, they are commodified and visualized to meet the demands of mass digital consumption.

### 4.2 The joint influence of athletes’ individual expression, self-expression desire, and idol worship group psychology

Personality, as a psychological concept, manifests as differences in psychological outlook and attitudes and behavior patterns toward the social environment. In sociology, personality is studied from the logical structure, the process of individualization, and social laws, referring to differences caused by different professions. According to philosophical research paradigms, personality ranges from rational idealism (Descartes’ “I think, therefore I am” mental self—personality, Kant and Hegel’s self—consciousness personality), irrationalism (Schopenhauer’s will to live, Nietzsche’s will to power), to the “existence” personality of inner subjective experience and feelings (Heidegger and Sartre’s spiritual personality, desire personality), Freud’s “libido” biological instinctual impulse personality, and Marx and Engels’ critique of Feuerbach’s individual metaphysical humanism, exploring the construction and development of personality in the relationship between natural and social existence (Gong, 2013).

Since the inception of the ancient Olympic Games, competitive sports have been a venue for athletes to freely express and release their personalities. The natural existence of personality is manifested in the pursuit of physical strength and the primal impulse triggered by the subjective experience of success and pleasure. In the social existence relationship, personality demands are expressed in the full display of individual value within the construction of social identity psychology. In the digital media environment, athletes with distinct personalities can create more media discourse and gain media attention. For example, the fearless Liu Xiang, the ascetic Su Bingtian, the rebellious Zhang Jike, and the adorable Sun Yingsha. Athletes’ personality expressions, imbued with network public opinion cultural traits, quickly gain popularity on social media platforms, embodying the widespread network subculture and the full release of athletes’ self—expression desires in the media—empowered era. The information public opinion field environment provides a relatively open cultural space for athletes’ personality expression and self—expression desires. Athletes’ discourse power returns and reappears in the network space, especially with the presentation of athletes’ life personalities, changing previous stereotypes, and diminishing the mystery and halo of fame. This satisfies fans’ voyeuristic psychology, narrows the distance between the public and athletes, and facilitates the formation of network public opinion culture driven by group idol worship psychology.

### 4.3 Societal demand for sports public figures and the rise of new social networks

Since the 2008 Beijing Winter Olympics, China has hosted numerous major sports events, including the East Asian Games, Asian Youth Games, Youth Olympic Games, World Athletics Championships, Military World Games, Universiade, Beijing Winter Olympics, and the Asian Games. During the organization and preparation of these events, athletes, as “scarce” information resources in network news reports, have attracted nationwide attention and have been widely disseminated on social media platforms. Sports have become a hot topic of widespread social concern. Particularly after sports were given a thematic focus, various sports news information has spread across cyberspace. Additionally, individuals freely publish news information through self-media, making sports-related information more complex and diverse.

To mine highly attractive resources from the vast amount of online public opinion information, some professional online public opinion incubation platforms have taken individuals with high societal demand for sports public figures as incubation targets. They systematically plan and package athletes, leveraging the advantage of “scarce” information resources to gain popularity on the internet and remain active on major online media and social platforms. Under the scrutiny of frequent exposure and fan frenzy in the culture of online public opinion dissemination, societal demand for sports public figures is no longer limited to their performance in competitions. The social role of athletes’ online identities increasingly involves educating, regulating, and guiding youth behavior, and creating a positive sports social culture environment.

In the digital media era, the booming development of the internet industry has replaced traditional offline sports fan clubs with new online social networks. The dissemination of competitive sports public opinion online represents a new form of media with digital attributes assigned to traditional print media. The rise of new social networks has forcibly intervened in the development of the competitive sports culture dissemination industry. Various online production and consumption behaviors surrounding sports news reporting have driven the generation of online public opinion culture. For example, the February 4, 2024, friendly match between the Major League Soccer team and the Hong Kong Star Team saw a collective online outcry when Messi did not play. The Hong Kong SAR government issued two statements expressing “extreme disappointment,” and top global media reported on the incident under the theme of “deception,” sparking widespread online debate. Against the backdrop of societal demand for sports public figures and the rise of new social networks, the public freely forms social groups with common idols in virtual interactive spaces. They tend to choose and focus only on information that they desire or find pleasing, while showing strong rejection of other information. In this social network interaction logic constructed by the online public opinion dissemination mechanism, long-term reception of homogeneous information occurs, gradually reinforcing stereotypes of athletes. This not only disconnects from reality but also easily leads to extreme ideas, causing competitive sports to enter the “information cocoon” crisis in the new social network space (Sunstein, 2008).

## 5 Strategies for purifying the space of competitive sports public opinion dissemination in the digital media environment

In light of the preceding phenomenological interpretation of online public opinion dissemination in competitive sports, the analysis of its symptomatic misconduct, and the attribution of such behaviors, a critical question arises: how can a clean, orderly, and virtuous public opinion environment be cultivated for competitive sports in the digital media era? The concept of purification—often discussed in philosophical and psychological domains—refers to the process of eliminating internal impurities, desires, or negative emotions to achieve spiritual clarity and elevation. Hegel referred to it as a “spiritual uplift” that transcends the natural state. In sociocultural contexts, purification denotes the removal of undesirable or harmful elements in order to restore clarity and order, emphasizing a transition from chaos to structure (Zhang, 1999). Building on this understanding, the present study proposes a set of targeted solutions from the perspective of purifying the online public opinion space. These strategies aim to reestablish a rational and constructive environment for the dissemination of competitive sports discourse in the digital sphere.

### 5.1 Moral constitutional governance: strengthening the construction of network public opinion morality and legal systems

The widespread dissemination of competitive sports news information in the digital media environment, due to the lack of moral and legal constraints, coupled with the inadequacy of existing criminal and tort laws in addressing public opinion violence and infringement, has led to the rampant spread of negative online public opinion behaviors such as ranking support, volume brushing, malicious hype, abusive attacks, rumor fabrication, and public opinion manipulation. The severity of these public opinion moral system issues has indeed reached a critical moment for “constitutional governance.” During the 2024 National People’s Congress in China, many representatives and experts advocated for the introduction of the “Anti—Cyber Violence Law” to enhance legal awareness and rights protection concepts in network public opinion ecological governance. This requires establishing higher—level legislative demands to complete the overall regulation of the network moral system—moral constitutional governance—to prevent capital’s forced intervention from corrupting network public opinion morality (e.g., internet trolls, paid post deletions). Specifically, the opinions of representatives and experts from the National People’s Congress can be adopted to introduce the “Anti—Cyber Violence Law” as the written constitution of the network public opinion moral system. However, due to the complexity of network public opinion dissemination, moral constitutional governance may find it challenging to timely identify and restrict infringing information. Additionally, there is no effective technical monitoring rule or standard for restricting cyber violence speech. For example, using numbers or letters to spread negative public opinion information to cover up infringement acts, but these symbols’ network language meanings already have widespread dissemination attributes and can cause substantial harm to others, should be included in the scope of infringement. The “Anti—Cyber Violence Law,” as an important legal basis for the moral constitutional governance of competitive sports network public opinion, also requires the establishment of a specialized Sports Network Ethics Committee. This committee, utilizing tools such as artificial intelligence and algorithms, can restrict and delete cyber violence speech in the field of competitive sports.

### 5.2 Emotional guidance: mainstream media leading the healthy and orderly development of network public opinion

In the field of competitive sports, the idol worship group psychology often exhibits strong emotional exclusivity, making emotional outbursts or polarization likely in network public opinion dissemination. This necessitates the emotional guidance role of mainstream media in network public opinion (Mutz, 2001; McCombs and Valenzuela, 2020). Mainstream media platforms, as representatives of network public opinion discourse dissemination, possess high authority, audience base, and media credibility. These platforms guide the development direction of network public opinion by publishing sports news, short videos, or live broadcasts. Mainstream media hold the comparative advantage of being the first to release and edit sports news information, making them an important medium for establishing the relationship between “official public opinion” and “public opinion.” The collision of official discourse transmission and public emotional expression on mainstream media platforms plays a crucial role in leading the healthy and orderly development of network public opinion. To address the issue of emotional disorder in the empathic dissemination of competitive sports news in the network space, it is necessary to leverage the emotional guidance role of mainstream media in competitive sports network public opinion. Specifically, experts or sports stars can be invited to “live interactive broadcasts” to clarify public misunderstandings about public opinion events and clarify facts. Alternatively, cultivating forum “opinion leaders” who can deeply analyze, rationally, and objectively judge network public opinion can help establish a “large public opinion field” that rationally and consciously guides the correct direction of public opinion development.

### 5.3 Collaborative governance: constructing mechanisms for collaborative network public opinion dissemination

Due to the influence of a free, open, and tolerant network public opinion dissemination space, the disorder caused by hegemonic public opinion disrupts the relationship within the community of interests, leaving it in a loose, unorganized, and chaotic state (Li and Zhang, 2016). Therefore, leveraging the advantages of collaborative governance to construct a community of interests will become an effective path for governing the dissemination order of competitive sports network public opinion. The collaborative governance mechanism for competitive sports network public opinion needs to be developed from the following aspects:

First, Collaborative Dissemination Subjects: Given the diversity of stakeholders in competitive sports and the complexity of their internal relationships, as well as the evolving characteristics of new social network spaces into arenas of multi—interest games, it is essential to establish a network public opinion dissemination interest collaboration chain among professional sports associations, clubs, practitioners (athletes, coaches, referees, managers), fans, broadcasters, sponsors, local governments, and intermediaries. The goal is to standardize the information dissemination behavior of competitive sports stakeholders, making it orderly and aligned with the network public opinion dissemination community of interests, and to construct new bond relationships based on interest demands.

Second, Collaborative Dissemination Platforms: Considering the diversity of platforms for disseminating competitive sports network public opinion information, the lack of inter—platform connections, and the absence of a cross—platform matrix structure, different platforms may present varying, even opposing, information on the same sports news. This information disparity can easily lead to conflicting viewpoints. Therefore, from the perspective of collaborative dissemination platforms, it is necessary to create a high—quality, precise integrated service full—media matrix to guide the correct direction of network public opinion.

Third, Collaborative Dissemination Content: Collaborative dissemination of competitive sports network public opinion content does not emphasize consistency but rather exclusivity and differentiation. Faced with the vast amount of information in network public opinion, many competitive sports news reports differ only in titles, with much of the content being similar or causing misunderstandings due to unprofessional expression. Therefore, from the perspective of collaborative dissemination content, it is necessary to process, arrange, combine, frame, and edit the news materials provided by frontline reporters of competitive sports events, supplemented by images, sounds, and actions to form terminal news products, thereby creating the correct public opinion guidance.

## 6 Conclusion and discussion

In the digital media environment, the dissemination of public opinion concerning competitive sports functions as a double-edged sword. On the one hand, it serves as a vital channel for generating information dividends, enhancing the visibility and societal influence of competitive sports, and stimulating digital sports consumption. On the other hand, the absence of adequate moral and legal regulation, the excessive commercialization of athletes’ public profiles, and the prevalence of utilitarian thinking have led to serious issues. These include digital public safety risks, severe disruptions to athletes’ daily lives, training, and competition routines, and even chaotic loss of control in the information dissemination process. A prominent manifestation of this distortion is the idolization of “internet celebrity” athletes who are portrayed as heroic figures regardless of performance outcomes, while their opponents become frequent targets of online abuse. Such misrepresentations pose significant barriers to the sustainable and healthy development of competitive sports. From the perspective of Actor-Network Theory (ANT), the root causes of these phenomena can be attributed to both individual-level and societal-level dynamics. On the individual level, athletes engage in role-playing, personalized expression, and the pursuit of self-presentation. On the societal level, the rise of digital consumer culture, the psychology of idol worship, and the public’s demand for sports celebrities serve as driving forces behind these issues. Therefore, from the integrated perspectives of moral constitutional governance, emotional guidance, and collaborative co-governance, this study proposes targeted solutions to the dissemination problems and their underlying causes in the digital media environment. Specifically, the research advocates for the development of ethical and legal frameworks for managing online discourse, strengthening the guiding and supervisory role of mainstream media, and establishing a collaborative governance mechanism involving multiple dissemination actors. From the perspective of the practical dissemination landscape, the generation and evolution of public opinion in the competitive sports field are rooted in the public’s value recognition and their acceptance of digital network order. The fertile ground for this phenomenon lies in the traffic value generated by the network economy and the algorithm-driven transformation of public opinion in the digital age. However, in an open, free, and tolerant online environment, the lack of effective information control and public opinion supervision has resulted in a situation where opportunities and risks coexist. In particular, the excessive hype surrounding professional athletes distorts public perception of sports and contributes to a climate of digital violence, thereby undermining the integrity of competitive sports. The internet, as a mirror of virtual reality, has become the most dynamic form of social space today. The “subcultural” phenomena emerging from online public opinion surrounding competitive sports must be critically examined. It is essential to regulate the online behavior of professional athletes, protect public image, and strengthen supervision and guidance mechanisms for digital public opinion, in order to foster a clean, rational, and constructive communication environment that promotes the high-quality dissemination of competitive sports culture.
